# An update on brown adipose tissue and obesity intervention: Function, regulation and therapeutic implications

**DOI:** 10.3389/fendo.2022.1065263

**Published:** 2023-01-11

**Authors:** Xiaomeng Liu, Zhi Zhang, Yajie Song, Hengchang Xie, Meng Dong

**Affiliations:** ^1^ Institute of Translational Medicine, College of Life Science and Agronomy, Zhoukou Normal University, Zhoukou, Henan, China; ^2^ Department of Nutrition and Food Hygiene, College of Public Health, Xinxiang Medical University, Xinxiang, Henan, China; ^3^ Key Laboratory of Animal Ecology and Conservation Biology, Institute of Zoology, Chinese Academy of Sciences, Beijing, China; ^4^ School of Life Science and Technology, ShanghaiTech University, Shanghai, China

**Keywords:** obesity, brown adipose tissue (BAT), thermogenesis, uncoupling protein1 (UCP1), browning

## Abstract

Overweight and obesity have become a world-wide problem. However, effective intervention approaches are limited. Brown adipose tissue, which helps maintain body temperature and contributes to thermogenesis, is dependent on uncoupling protein1. Over the last decade, an in-creasing number of studies have found that activating brown adipose tissue and browning of white adipose tissue can protect against obesity and obesity-related metabolic disease. Brown adipose tissue has gradually become an appealing therapeutic target for the prevention and re-versal of obesity. However, some important issues remain unresolved. It is not certain whether increasing brown adipose tissue activity is the cause or effect of body weight loss or what the risks might be for sympathetic nervous system-dependent non-shivering thermogenesis. In this review, we comprehensively summarize approaches to activating brown adipose tissue and/or browning white adipose tissue, such as cold exposure, exercise, and small-molecule treatment. We highlight the functional mechanisms of small-molecule treatment and brown adipose tissue transplantation using batokine, sympathetic nervous system and/or gut microbiome. Finally, we discuss the causality between body weight loss induced by bariatric surgery, exercise, and brown adipose tissue activity.

## 1 General overview of obesity and BAT

The increasing overweight and obesity pandemic has received significant attention worldwide. A total of 108 million children and 604 million adults are currently considered obese all by the year 2015 (1). The body mass index report of 31.5 million adults worldwide from 1975 to 2016 shows that the prevalence of overweight increased from 26.6% to 39%, and the prevalence of obesity increased from 7% to 12.5% (2). Obesity is associated with a shortened lifespan as well as with various types and degree of risks, such as insulin resistance and type 2 diabetes mellitus (T2DM), hyperlipidemia, stroke, and cardiovascular disease (3). Keaver et al. reported that on current trends, the proportion of overweight or obese people in Ireland could reach 85% by 2030. Obesity-related diseases such as cardiovascular disease increased by 97%, cancer by 61% and type 2 diabetes by 21% (4). The rapidly increasing prevalence and disease burden of elevated BMI highlights the need for a continued focus on ways to fight obesity. Obesity develops from excessive food intake or inadequate total energy expenditure, including basic energy expenditure, activity-based energy expenditure, diet-induced thermogenesis, and energy expenditure from thermoregulation. For this reason, caloric restriction and increased exercise are the most common ways that most people lose weight over a long period of time. Although these are effective, dieting and exercise must be maintained for a long time, and the risk remains that body weight will return. Bariatric surgery and drugs have also been used to treat obesity (5). However, bariatric surgery carries a high risk of considerable morbidity and potential mortality (6). As of 2020, the following were FDA-approved anti-obesity drugs: Orlistat, Lorcaserin, Phentermine/Topiramate, Naltrexone/Bupropion, and Liraglutide (7). Over the last few decades, several anti-obesity drugs have been withdrawn from the market due to their side effects. Sibutramine, for example, increases the risk for heart attack and stroke. The use of 2,4-dinitrophenol increases the risk for neurological diseases and cataracts, and when used in high doses, it will lead to irregular respiratory uncoupling of all cells, resulting in high temperature and death (8, 9). Sibutramine have been withdrawn owing to hepatic injury, Orlistat has some unacceptable side effects (10). Lorcaserin increases the risk of breast cancer (11).

Obesity develops form excessive energy intake exceeds energy expenditure over a long period. In addition to white adipose tissue (WAT), which has the function of storing excess energy intake, the body also has an energy-consuming adipose tissue, brown adipose tissue (BAT), which heats the body and stores thermal energy. Compared with white adipocytes, brown adipocytes have more lipid droplets but smaller size, and have more mitochondria (12). In the early days of the study, brown fat was believed to be widely present in many species during infancy, including humans. With the development of technology and concept, in 2009, people intuitively observed the existence of active brown fat in adults through 18 fluoro-deoxy-glucose positron emission tomography coupled with computed tomography (18F-FDG-PET/CT) (13). As a necessary thermoregulator in early life, BAT are present in a large number in infants and children. In adults, the number of BAT at the scapula basically disappears, and there are a few active BAT at the clavicle, pericarotid artery and pericardium (13–15). With the increase of age, the probability of BAT detection in human body decreases. Using PET/CT scans of subjects’ BAT, results showed that BAT detection rates were three times higher in subjects under 50 than in subjects under 64 (16). In addition to age, the distribution of BAT in the body of men and women is also different. PET/CT scan results show that the detection rate and content of BAT in the body of females are higher than that of males (16).

**Figure 1:**
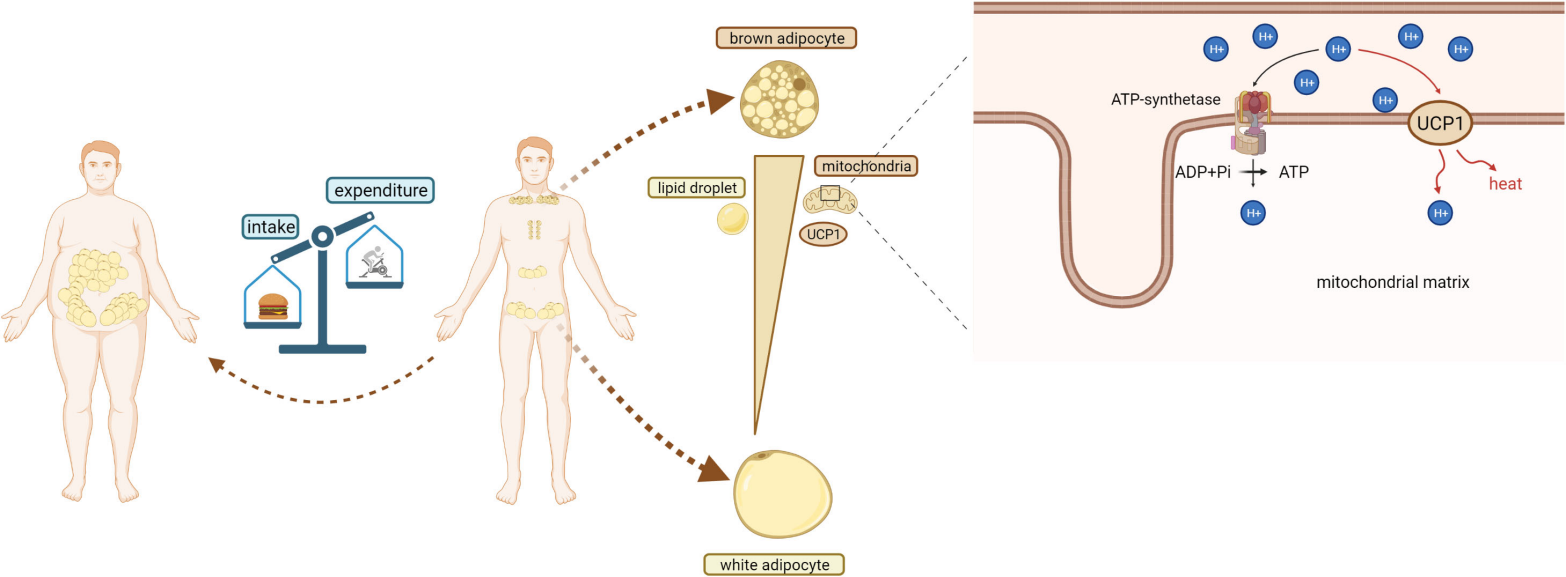
Obesity and BAT.Obesity occurs when the body’s energy intake is greater than its energy consumption. In adults, BAT is found in the back scapula and clavicle area, around the heart and kidneys, and WAT is located around the viscera and groin. Compared with white adipocytes, brown adipocytes contain a large number of mitochondria and high expression of UCP1. Brown adipocytes also has a small volume and a large number of lipid droplets. In mitochondria of brown adipocytes, UCP1 mediates protons to pass through the inner membrane of mitochondria, dissipating proton (H+) gradient and generating heat.

**Figure 2:**
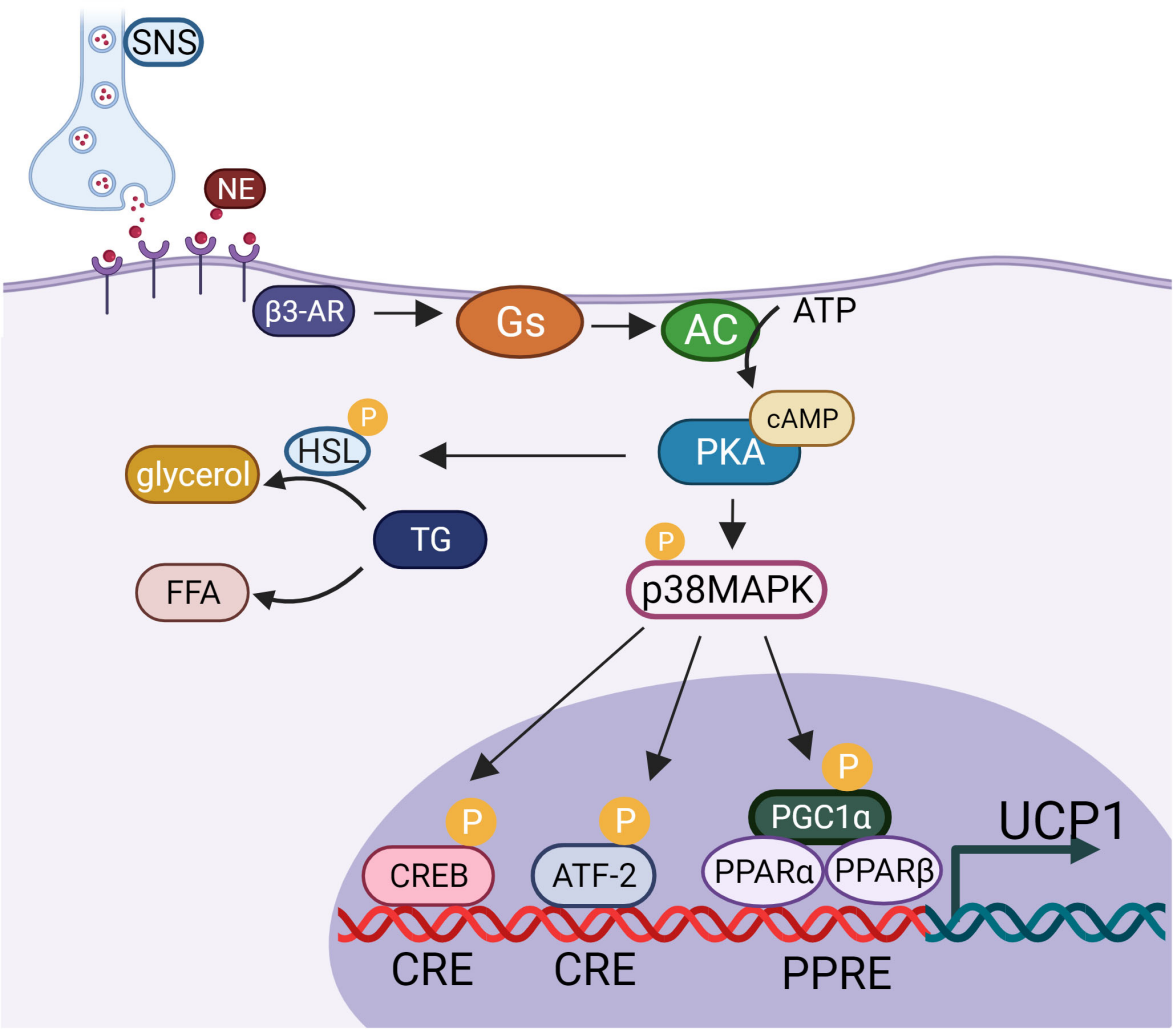
UCP1 activation pathway.SNS, sympathetic nervous system; NE, noradrenaline; β3-AR, β3- adrenergic receptor; Gs, guanylate binding protein; AC, adenylate cyclase; cAMP, cyclic adenosine monophosphate; PKA, protein kinase A; HSL, hormone-sensitive triglyceride lipase; TG, triglycerides; FFA, free fatty acids; p38MAPK, p38 mitogen activated protein kinase; CREB, cAMP response element binding protein; CRE, cAMP response element; PGC1-α, PPARγ coactivator-1α; ATF-2, activating transcription factor- 2; PPAR, proliferator-activated receptor; UCP1, uncoupling protein1.

The thermogenesis of BAT depends on uncoupling protein1 (UCP1), a fatty acid anion transporter present in the mitochondrial inner membrane. UCP1 uncouples the oxidative respiratory chain of mitochondria that prevents ADP from synthesizing ATP, resulting in the release of energy in the form of thermal energy, which is directly governed by sympathetic nerve fibers (**Figure 1**). In mature adipocytes, noradrenaline (NE) released from sympathetic nervous system (SNS) binds to β3-adrenergic receptor (β3-AR) to promote guanylate binding protein (Gs) activates adenylate cyclase (AC). Activated AC can convert intracellular ATP into cyclic adenosine monophosphate (cAMP), increase intracellular cAMP concentration, and activate cAMP dependent protein kinase A (PKA), which activates hormone-sensitive triglyceride lipase (HSL) and accelerates the hydrolysis of triglycerides (TG) to glycerol and free fatty acids (FFA). At the same time, PKA can also activate p38 mitogen activated protein kinase (p38MAPK), and then activate the downstream substrate PPARγ coactivator-1α (PGC1-α) and activating transcription factor-2 (ATF-2). On the one hand, activated p38MAPK promote the phosphorylation of PGC1-α, and activated PGC1-α binds to the UCP1 promoter through the coactivator peroxisome proliferator-activated receptor (PPAR) to jointly promote the transcription of UCP1. On the other hand, activated p38MAPK phosphorylates ATF-2, and activated ATF-2 promotes the transcription of UCP1 through cAMP response element binding protein (CREB). PKA can also directly phosphorylate CREB. After activated CREB binds with cAMP response element (CRE), it can directly induce the expression of UCP1 (17, 18) (**Figure 2**). Males have a higher active BAT ratio than females, and its activity gradually decreases with age. In addition, the activity of brown fat is also significantly reduced with the development of obesity (19–23). It is estimated that when BAT is fully activated, only 50 g BAT can consume 20% of the body’s basal metabolic energy (20). Studies found that under cold or β-adrenergic agonist stimulation, brown-like adipocytes, known as beige cells, appeared in the subcutaneous WAT of mice. Beige adipocytes have multilocular fat droplets, high mitochondrial content and expression of UCP1. Beige adipocytes also have a thermogenic function, which can promote energy consumption. Its role in anti-obesity has attracted more and more attention. Trying to efficiently use brown or beige adipocytes in the human body to burn energy to reduce body fat content has been become one of the breakthrough points worth looking forward to in the current anti-obesity field (24).

## 2 Anti-obesity function of BAT

Based on the powerful energy-expenditure function of adaptive thermogenesis in the human body, BAT can be used as a target to reverse obesity and obesity-related metabolic diseases. The hope of identifying the anti-obesity function of BAT and establishing ways of activating it has aroused intense enthusiasm among scientists (25, 26). In recent decades, a large number of studies in different model organisms have highlighted the importance of the anti-obesity function of BAT through increasing energy expenditure (27–29). In addition to its thermogenic functions, BAT can also regulate energy metabolism of the whole body in model animals by secreting batokine through the autocrine and paracrine methods (30, 31). This review summarizes recent advances in methods of activating BAT and WAT browning in the past decade, such as cold exposure, exercise, diet, small-molecule treatment, and BAT transplantation. To illustrate the potential efficiency of these reagents in activating BAT or in WAT browning to thermogenesis, related molecular mechanisms are also elucidated. We also investigate the relationship between BAT activity and weight loss and examine means of optimizing the pathway of BAT activation, as well as reporting a few advances.

## 3 Cold-exposure: The most classic way to activate BAT

For humans and rodents, BAT is a thermogenic tissue whose main function is heat production *via* non-shivering thermogenesis (NST) when activated by cold exposure (26–28). In response to this exposure, NE released from the SNS regulates brown adipocytes at multiple levels, and it binds to β3-AR on brown and beige adipocytes. Subsequently, UCP1 becomes highly expressed and activated, thereby promoting lipid β-oxidation and heat production (32). These steps promote the proliferation and differentiation of brown preadipocytes to increase the level of NST (33–35). It is worth noting that increased sympathetic excitability can cause an accelerated heartbeat. Interestingly, other mechanisms also exist that regulate this. Li (36) found that gut microbes play a key role in BAT activation with cold exposure. A short-chain fatty acid (butyrate) produced by gut microbes appears to be an important component in the activation of BAT during cold exposure. In addition, low ambient temperatures can also induce WAT browning in a UCP1-dependent manner (37). The researchers found that the products of 12-lipoxygenase in mouse serum increased under cold stimulation, and 12-lipoxygenase in brown adipocytes produced related lipoxygenase and promoted the uptake and utilization of glucose by fat and muscle, while the products of 12-lipoxygenase in obese individuals decreased significantly (38). At present, studies of BAT activation have focused mainly on exposing rodents to severe cold (typically 4–10°C) (34, 39). Obviously, this kind of cold exposure is not practical for use against human obesity. In fact, the temperatures (21–22°C) found in standard animal facilities in these studies are already much lower than rodent’s thermal neutral zone of 30°C, which profoundly impacts their basal metabolic rate, and some scientists predict that this temperature might already prompt BAT to consume energy (37). Interestingly, this view has been confirmed by some recent findings that show mild cold (20°C or 22°C) exposure also significantly increases the thermogenic function of BAT (40, 41). These provides us with a new strategy for anti-obesity possibilities of human BAT, namely, that mild cold exposure can be used in place of severe cold exposure. In addition, it is important to recognize that atherosclerosis patients can lose weight and increase their risk for cardiovascular events by activating brown fat. The faster the heartbeat, which is directly related to the sympathetic nerve, the greater the cardiovascular mortality (42). Traditionally, beige fat is considered to have browning potential in cold environment, which can promote heat production and energy consumption be in cold or cold weather (43). Recently, scholars have found that in addition to cold exposure, beige fat can also sense local mild thermal effects (local hyperthermia therapy, LHT) and activate thermogenesis through heat shock transcription factor 1 (HSF1). Rather than the traditional view of cold activation of beige fat, LHT achieved using a hydrogel-based photothermal therapy activated beige fat, it is worth noting that the weight loss caused by local hyperthermia therapy will not be accompanied by the change of NE in blood (44).

## 4 Exercise-induced browning of WAT

In 1991, Stallknecht et al. first indicated that swimming training increases the mitochondrial enzyme activity of WAT in rats and gives a mitochondria-enriched fraction of WAT a browner appearance (45). However, the specific mechanism of the browning of WAT has not been identified. In 2012, Boström et al. identified a new myokine, irisin, which is released into the circulation during exercise and triggers the transformation of white fat cells into brown-in-white cells in mice (46). However, Norheim et al. found that, after 12 weeks of training in humans, skeletal muscle mRNA for PGC1-α and fibronectin type III domain-containing protein 5 (FNDC5) mRNA levels increased, and surprisingly, circulating irisin was reduced. UCP1 mRNA did not correlate with FNDC5 expression in subcutaneous adipose tissue or skeletal muscle (47). Except for irisin, other exercise-induced circulating factors such as Catecholamines (48), Interleukin-6 (IL-6) (49), Meteorin-like protein (50), fibroblast growth factor 21 (FGF21) (51) also have connection with exercise-induced browning of WAT (52). The development and maintenance of the brown phenotype in adipose tissue is maintained by SNS and noradrenergic post-ganglionic neurons. A massive activation of the SNS was observed during exercise, in addition to swimming training, running training can counteract obesity by an adrenergic-regulated brown recruitment of adipocytes, and increases adipose progenitor cell population in BAT to ameliorate high-fat diet-induced metabolic and vascular dysfunction (53, 54). These findings, as well as others (46, 54, 55), have demonstrated that exercise training such as swimming, voluntary wheel running and treadmill running leads to the browning of rodents WAT in a variety of ways. After moderate exercise, the sympathetic nerve activity increased and the expression levels of β3-AR and UCP1 were up-regulated in BAT of early overfed male wistar rats, then resulting in increased thermogenic function of BAT and higher energy consumption (56). However, exercise training consistently fails to induce browning in humans even though exercise brings other benefits to human’s health (47, 57, 58). Surprisingly, a recent randomized and controlled study found that after combined training, the thermogenic activity of BAT was significantly increased in overweight or T2DM patients, and the expression of genes related to thermogenic profile (TMEM26, EPSTI1) in subcutaneous fat was significantly increased (59). However, some experimental evidence in rats suggests otherwise. Sedentary rats exposed to cold had higher amounts of total protein and DNA in brown adipose tissue than those in the exercise group. It may be that exercise leads to the heat production of muscles, while reducing the heat production of BAT (60). Although a large body of evidence shows that exercise increases browning of WAT, whether it increases heat production is still debatable.

## 5 Dietary patterns

Previous studies have shown that food intake can effectively activate the thermogenesis of BAT in humans and rodents (61, 62). Researchers found that post-prandial glucose surge and increased insulin affect the transcription of clock gene, BAT activity exhibits glucose-dependent circadian rhythm (62), this concept retains for quite a while. To our surprise, another group of researchers has also recently identified that only insulin, not glucose, can rapidly induce the expression of PERIOD, which is a clock gene, and that insulin and IGF-1 are primary signals of the cellular clock feeding time (63).

In addition, certain special dietary patterns can induce the browning of WAT and BAT thermogenesis through different mechanisms. CDC-like kinase 2 (CLK2) responds to high fat diet (HFD) and is expressed in BAT, is upregulated upon refeeding, and then enhances CREB-dependent UCP1 expression (64). Moreover, every-other-day fasting (EODF) (one day feeding-one day fasting) for 15 cycles can selectively upregulate monocarboxylate transporter 1 expression in beige cells by shaping the gut microbiota. EODF stimulates beige fat development within WAT and dramatically ameliorates obesity, insulin resistance, and hepatic steatosis (65). Intermittent fasting (IF) (one day fasting-two days refeeding) for 12 weeks can activate BAT through pregnancy zone protein (PZP) secreted by the liver, so as to promote diet induced heat generation, and finally have the effect of anti-obesity (66). In addition, WAT depots are smaller and denser in 60% calorie-restricted diet fed mice. caloric restriction (CR) leads to browning of WAT, promotes the development of functional beige fat, and enhances the type 2 immune response and silent information regulator type 1 (SIRT1) expression (67). These dietary patterns seem to be more appropriate for anti-obesity treatment than stimulation of the SNS, such as through the action of the gut microbiota, and E. faecalis and its metabolite MA can reduce adiposity through BAT activation and beige fat formation (28). In summary, changing dietary patterns may be a relatively safe and feasible method of browning WAT as an anti-obesity measure. Considering the synergy between different dietary restrictions, we speculate that the insulin rhythm caused by the feeding behavior of mice is consistent with the observation that the circadian rhythm of BAT can activate BAT.

## 6 Small molecules

Modern studies have highlighted the potential function that some small molecules in fruits and vegetables have in browning of WAT, activating BAT, and preventing and treating obesity (**Table 1**). Long-term consumption of certain plant extracts, such as capsaicin (68, 90) and its esters, ephedrine (75, 91), and green tea (92) can directly activate brown fat, thereby increasing energy consumption and achieving weight control (75). The Ephedrine in *ephedra sinica Stapf* can increase the energy metabolism of BAT (93), and the active ingredient in red peppers, capsaicin may enhance the metabolism by directly or indirectly activating the β-adrenergic pathway (94, 95). Capsaicin has a strong initial oral effect, and its local administration is irritating, which limits its clinical application. Urolithin A is a major microbial metabolite derived from polyphenolics of berries and pomegranate fruits (96), it has also been shown to inhibit HFD-induced by activating the ability of brown and beige fat to produce heat in mice, which is dependent on the thyroid hormone pathway (97). Resveratrol, a polyphenolic compound rich in a variety of plant species, has been found to affect the expression of SIRT1, PGC1-α, and AMPK to improve mitochondrial function and promote BAT production (73, 98, 99). Recently, it has also been found that it can also regulate bile acid metabolism through gut microbiota remodeling, so as to activate BAT and promote WAT browning (100). The mulberry grows widely, and its extracts anthocyanin 3-O-glucoside (C3G) and rutin (Rut) are widely used in daily life (31, 87). C3G activates AKT, ERK, and p-38 signaling pathways in subcutaneous fat and epididymal fat. At the same time, Rut upregulates the ERK signaling pathway in subcutaneous fat and the AKT signaling pathway in muscle to improve insulin sensitivity (31, 87). Hypericin (HPF) extracted from *Hypericum perforatum* can directly target dihydrolipoamide S-acetyltransferase to regulate AMPK-PGC1-α signal pathway, and finally upregulate the expression of UCP1, promote adipose tissue heat production (88). Large-leaf yellow tea extract can prevent the reduction of these genes that is induced by HFD (UCP1, PGC1-α) in subcutaneous adipose tissue (SAT) and BAT, promote the beige of SAT and activate the thermogenic function of BAT (89). Ephedrine may cause side effects, such as increased heart rate and blood pressure, and may increase the number of metabolites in the circulatory system, but it does not activate brown fat in adults (101). There have been few reports on effects of resveratrol on body weight in human trials (102–104). Although the possibility that rutin regulates BAT metabolic activity through stimulating the SNS cannot be excluded (105), cellular experiments have indicated that rutin directly activates BAT oxidation *in vitro*. Several dietary compounds have been shown to activate brown fat activity at clinical levels (**Table 2**), and increasing evidence has revealed that thermogenic regulators have therapeutic effects against obesity through increasing BAT mass and/or activity, and some products have been involved in phase 1, 2, 3, and 4 clinical trials, such as β3-AR agonist and fluvastatin (112, 113). Endogenous molecules, such as irisin and FGF21, might be potential targets for certain molecules. However, the risks of these molecules to the central nervous system and sympathetic nerve activation should be considered (114).

**Table 1:** A dietary compound capable of activating BAT.

Dietary component	Source	Effect	Refs
**Capsaicin**	Hot pepper	Activating the TRPV1 channel, inducing browning of white adipose tissue	(68)
**Parviflora extract**	Kaempferia parviflora	Promoting energy metabolism by activating of BAT and up-regulating of UCP1 protein	(69)
**Thymol**	Aromatic plants (Thyme species)	Promoting mitochondrial biogenesis and enhancing expression of a core set of brown fat-specific markers as well as increasing protein levels of PPARγ, p-AMPK, PGC1-α, and UCP1	(70)
**Chrysin**	Flowers Mushroom	White fat browning is increased by the AMPK-mediated pathway	(71)
**Curcumin**	Turmeric	Increasing gene expression levels (UCP1, PGC1-α, Cidea, Prdm16 and Elovl3) and plasma norepinephrine concentrations	(72)
**Resveratrol**	Red cabbage Red wine Berries	Increasing UCP1 and SIRT1 expression	(73, 74)
**Tea catechins**	Cocoa Oolong Pu-erh	Enhancing the mRNA expression of UCP-1 in rat BAT	(75)
**EPA, DHA**	Fish oil	Reducing the accumulation of fat, improving the metabolism of mice, and increasing the expression of UCP-1	(76)
**Berberine**	Coptis chinensis Hydrastis canadensis	Inducing the development of inguinal brown-like adipocytes, and increasing the expression of heat source markers PGC1-α, Cidea, and UCP1	(77)
**Pungency: paradol**	Ginger	Increasing whole-body energy expenditure through the activation of BAT	(78)
**Quercetin**	Broccoli Berries Asparagus	Inducing the expression of BAT-specific genes and increasing the expression of carnitine palmitoyl transferase 1α	(79)
**Caffeine**	Coffee	Activating BAT thermogenesis and increasing metabolism	(80)
**Carotenoid: fucoxanthin**	Edible seaweed (Undaria pinnatifida)	Regulating cytokine secretion in WAT, improving insulin resistance, and decreasing blood glucose levels	(81)
**Allicin**	Garlic, Onion	Enhancing expression of brown adipocyte-specific genes and lipid oxidation	(82)
**Ginsenoside**	Panax ginseng	Reducing lipid droplets, stimulating UCP1 staining, and increasing expression of thermogenic and mitochondrial genes	(83)
**Rubi Fructus extract**	Rubus coreanus	Upregulating expression levels of thermogenic- and mitochondria-related genes	(84)
**Menthol**	Mint	Menthol activates the TRPM8 channel by mimicking cold exposure, upregulates UCP1 expression, and activates BAT to treat and prevent diet-induced obesity	(85)
**Green tea extract**	Green tea	Reducing the adipocyte size in the WAT and the lipid droplet size in the BAT, inducing the browning in WAT	(86)
**C_3_G**	Mulberry	Activating AKT, ERK and p-38 signaling pathways in subcutaneous fat and epididymal fat to improve insulin sensitivity	(87)
**Rutin**	Mulberry	Activating BAT by upregulating the ERK signaling pathway in subcutaneous fat and the AKT signaling pathway in muscle	(31)
**HPF**	Hypericum perforatum	Regulating AMPK- PGC1-α signal pathway, and upregulating the expression of UCP1 in WAT and BAT	(88)
**large-leaf yellow tea extract**	large-leaf yellow tea	Increasing mitochondrial copy numbers, and the expression of thermogenic genes (UCP1, PGC1-α, etc.)	(89)

**Table 2:** Clinical trials of effects of dietary compounds on BAT.

Strategy	Subjects	Effects on BAT	Refs
**Capsinoids**	Healthy, Middle-Aged Adults	Increased BAT density	(106)
**Capsinoids**	Healthy adults	Increased EE, fat oxidation, and heat production in the cervical-supraclavicular regions	(107)
**Resveratrol**	First degree relatives of patients with T2DM	Unaffected 18F-FDG glucose uptake in BAT	(108)
**Berberine**	Mildly overweight patients with non-alcoholic fatty liver disease	Increased BAT mass and activity	(109)
**Ephedrine**	Lean humans	Increased BAT activity	(110)
**Grains of paradise**	healthy men	Increased BAT activity	(111)
**Kaempferia parviflora extract**	healthy men	Increased BAT activity	(69)

## 7 BAT transplantation and batokine

With the rediscovery of BAT in adult humans, study on increasing the thermogenic function of BAT has reached an exciting boom. Many studies have focused on increasing the amount of BAT. This can be accomplished by transplanting extrinsic BAT, which is an effective way to reduce body weight gain in mice and to enhance whole-body energy metabolism; unexpectedly, however, transplanted BAT almost totally loses thermogenic function but activates the thermogenesis of endogenous BAT (27, 115). Indeed, Stanford et al. transplanted adult BAT into the visceral cavity of diabetic mice and found that IL-6 levels became elevated, which in turn induced an increase in FGF21 expression and improved glucose tolerance (29).

BAT transplantation has been performed in diabetic mouse models of dietary and obesity-related pathology. In most of these studies, BAT transplantation has shown beneficial effects on the metabolic health of recipient mice (transBATation) were fed a HFD (**Table 3**), and it has great potential as an anti-obesity strategy. BAT transplantation enhances systemic energy metabolism in a mouse model of diet-induced obesity (27). Many studies have shown that BAT transplantation can affect the action of batokine. In one study, the transplantation of embryonic BAT into a streptozotocin-induced mouse model of type 1 diabetes reversed the symptoms of diabetes, reduced inflammation, and elevated adiponectin levels. It is worth emphasizing that these effects were independent of insulin, and the level of IGF-1 was significantly elevated in BAT-transplanted mice (32). A few years later, similar results were achieved using a non-obese diabetes (NOD) model, showing that BAT transplantation combined the action of multiple adipokines to establish a new equilibrium in the animal to control chronic glycemia (118). In 2012, Harvard researchers transplanted adult rat BAT into the visceral cavity of diabetic mice and found that IL-6 levels became elevated in the mice, which in turn induced an increase in FGF21 expression. When the BAT used for transplantation was obtained from IL-6-knockout mice, the improved metabolic profile was lost, but glucose homeostasis improved. This result indicates that BAT-derived IL-6 is required for the effects of BAT transplantation on glucose homeostasis and insulin sensitivity (29).

**Table 3:** The effects of experimental BAT transplantation.

Source	Recipient	Localization	Effect	Refs
**Embryonic mouse iBAT**	Streptozotocin-induced type 1 diabetes mice	Subcutaneous region	Glucose tolerance is normal, increased adiponectin and reduced tissue inflammation	(116)
**Adult mouse iBAT**	DHEA-induced PCOS rat	The s.c. space of the dorsal region adjacent to the endogenous BAT	Enhanced endogenous BAT activity and thereby increased the circulating adiponectin level	(117)
**Embryonic mouse iBAT**	Autoimmune-mediated T1D model	Subcutaneous region	Increased IGF-1 levels and decreased inflammation and glucagon levels	(118)
**Adult mouse iBAT**	HFD-induced obese mice	Visceral cavity	Improved glucose tolerance, increased insulin sensitivity and IL-6 levels	(29)
**Adult mouse iBAT**	HFD-induced obese mice	Adjacent to BAT	Improved whole-body energy metabolism, increased insulin sensitivity, and reversed preexisting obesity.	(115)
**Adult mouse iBAT**	ob/ob mice	Dorsal subcutaneous region	Adiponectin levels and oxygen consumption increase, and total body fat mass decreases.	(27)
**Adult mouse iBAT**	HFD-induced obese mice	Dorsal subcutaneous region	Enhanced systemic metabolic response and increased sympathetic activity.	(119)
**Adult mouse iBAT**	Hph-1 mice fed with HFD	Visceral fat regions	Impaired BAT function, and metabolic disorders in the body are improved, regulating systemic energy metabolism.	(120)

These findings indicate the role of some potential protein factors in BAT function. In ob/ob mice with BAT loss, the expression of IL-6 and FGF21 in mice does not increase while the level of adiponectin increases significantly (27). This may be caused by different locations of transplantations in mice and different receptors. BAT transplantation improves whole-body energy metabolism and ameliorates polycystic ovary syndrome (PCOS), and the transplantation of BAT into PCOS rats significantly stabilizes menstrual irregularity and improves systemic insulin sensitivity up to a normal level. It also activates endogenous BAT and thereby increases the circulating level of adiponectin, which plays a prominent role in whole-body energy metabolism and ovarian physiology (115, 117). These results demonstrate that BAT transplantation may reduce obesity and related diseases by activating endogenous BAT. In addition, they show that transplanted mice confer resistance to HFD-induced obesity *via* increases in whole-body sympathetic activity (119). However, in view of the differences of Batokine between human and mouse, it is necessary to discuss BAT plasticity from the perspective of human physiology (14). For example, in rodents, FGF21 is secreted from BAT in response to thermogenic activation, while in human brown adipocytes, FGF21 is nearly undetectable (121).

## 8 Remaining questions

### 8.1 BAT activity and body weight loss: Which is the cause, and which is the effect?

As described above, there are various ways that BAT activity or the browning of WAT can be improved (**Table 4**). These approaches are often associated with body weight gain and loss (124, 125). Body weight loss can lead to increased activity of BAT. For example, in a recent study on bariatric surgery, which is an effective way to lose weight in obese individuals, it increased BAT activity, such that 1 year after surgery, the positive rate of BAT activity increased to 50% (125). This study partially demonstrated that, under the studied circumstances, BAT activity enhancement may be the result of body weight loss. However, some experimental evidence in rats suggests that bariatric surgery reduces volume, oxidative metabolism, and thermogenic gene expression in interscapular brown adipose tissue in rats (126). Researchers have shown that exercise can promote BAT recruitment, regulate the expression of UCP1, and enhance the function of mitochondria in mice and rats; meanwhile, increased BAT activity is accompanied by reduced body weight gain (127, 128). In fact, without body weight loss, long-term training has little effect on the browning of human subcutaneous adipose tissue, and it does not enhance the levels of UCP1 and PGC1-α (47). The same question concerning the small molecules that activate BAT remains. For instance, in one study, after treatment with a mixture of ephedrine and methylxanthine, MSG-induced obese mice increased their energy consumption by 20%, weight loss by 25%, and fat loss by 75% (129). When resveratrol reduced body weight and promoted BAT function, the researchers also observed that resveratrol could activate the expression of SIRT1, AMPK, and PGC1-α (99).

**Table 4:** Different ways to improve the BAT activity or browning of WAT.

Strategy	(Major) Target(s)	Effects on Glucose Metabolism	Effects on Lipid Metabolism	Effects on BAT Activity	Effects on weight	Refs
**Cold-exposure**	Gut Microbiota	–	–	UCP1↑	↓	(36)
**Cold-exposure**	UCP1、adiponectin	–	Lipolysis↑	BAT activity↑	↓	(37)
**Cold-exposure**	SNS	–	Adiponectin↓	UCP1↑	→	(122)
**Swimming training**	Irisin	Insulin resistance↓	–	Browning of subcutaneous adipose tissue	↓	(45)
**Running training**	Adrenaline	–	–	UCP1/PGC1-α↑ The average size of lipid droplet of the brown adipocytes↓	↓	(52)
**Food intake**	–	Insulin sensitivity↑	Lipid metabolism related genes expression is increased	BAT thermogenesis↑	→	(61)
**HFD**	Clk2、UCP1	–	–	Tissue oxygen consumption and protein levels of UCP1↑	↓	(64)
**EODF**	Gut Microbiota	Insulin resistance↓	–	Beiging of WAT↑	↓	(123)
**IF**	PZP	Insulin sensitivity↑	–	UCP1↑	↓	(66)
**CR**	Type 2 immune	→	–	The development of functional beige fat↑ UCP1↑	↓	(67)
**Capsinoids**	β-adrenergic	–	–	–	↓	(68)
**Resveratrol**	SIRT1, PGC1-α, AMPK	Insulin resistance↓	–	Mitochondrial function↑	↓	(98)
**Rutin**	AKT, ERK, p-38 signaling pathways	Insulin sensitivity↑	–	BAT activity↑	↓	(31)
**BAT transplantation**	Endogenous BAT	Insulin resistance↓	Adiponectin↑	Endogenous BAT activity↑	↓	(27, 115)

↑:raise/improve ↓:down →:No change

These findings raise the question: Between BAT activity and body weight loss, which is the cause and which is the effect?

Some possible answers present themselves. For instance, in one study, 4 h of cold exposure significantly enhanced BAT activity (130, 131). Hanssen et al. found that short-term (10-day) cold acclimation improved insulin sensitivity in patients with type 2 diabetes mellitus without body weight change. PET-CT results demonstrated that 10 days of cold acclimation induced thermogenesis of BAT in the scapula (132). Li et al. showed that, with 6 days of EODF treatment, mice WAT was greatly browned when the body weight showed no change (123).

At the same time, *in vitro* studies can be an adjunct method in some cases. Ephedrine can stimulate BAT respiration through adrenergic receptors *in vitro* (133), rutin directly activates BAT oxidation *in vitro* (31). In addition, *in vivo* studies on the absence of non-shivering (UCP1-dependent) thermogenesis should be performed to ensure if BAT and/or browning WAT are essential for the anti-obesity effects of these treatments. Kalinovich et al. observed the effects of C12TPP on high-fat-diet-induced obese mice. After C12TPP treatment, the body weights were significantly reduced. When UCP1-KO mice were treated with C12TPP, this effect disappeared, and the effects of C12TPP on the body weight of obese mice did not depend on UCP1 (134). All in all, many aspects of this exciting subject remain largely unknown and clearly merit further investigation.

### 8.2 SNS-dependent non-shivering thermogenesis

Stimulation of sympathetic nerves can cause abdominal visceral cardiovascular contraction and heartbeat enhancement and acceleration. Many treatments to stimulate the thermogenesis of fat can work through the SNS. Cold exposure stimulates sympathetic nervous (SN) excitability to promote the browning process (32, 135), and exercise can significantly activate the SN (53, 54). In addition, many small molecules can also enhance the excitability of the SN (105). At the same time, considering that SNS plays vital role in multiple physiological metabolic processes, these methods of stimulating or enhancing sympathetic excitability may have negative effects on the body. For example, cold stimulation can increase the thermogenesis of BAT through the gut microbiota (36), warm environments inhibit brown fat heat production, which is controlled by the nervous system (136). But cold stimulation also activates sympathetic nerves and increases systolic blood pressure, leads to rapid heartbeat and cardiac strain, which ultimately increases the risk of cardiovascular disease (137, 138). Cold stimulation is not a good anti-obesity strategy for people with cardiovascular disease. Many regulators target GPCRs, TRP, or nuclear receptors, and some have undergone clinical trials in different phases (112, 113). Transient receptor potential (TRP) ion channels are transmembrane ion channels that allow cations to pass through the cell membrane non selectively. They can mediate calcium influx and high intracellular calcium level activates the expression of PGC1α/UCP1 and increases BAT thermogenesis (139, 140). TRP channels can be activated by a variety of stimuli. The capsaicin receptor TRPV1 is expressed in brown adipose tissue and its expression level is elevated during the differentiation of pre-brown adipocytes (141).. TRPV2 was also highly expressed in brown fat, and the expression of heat genes in BAT decreased after TRPV2 knockout (142). Menthol acts as a ligand for TRPM8, activating TRPM8 in brown and white fat and improving glucose tolerance (143). The factors that promote the browning of WAT can increase thermogenesis in different ways, such as by increasing the expression of UCP1 (82) and stimulating the melanocortin/corticotropin system (144). Calcium supplementation can increase the gene expression of UCP1 and increase the protein expression of brown adipocytes marker genes, such as PGC1α, PDH, and Cyto C in inguinal WAT (145) (140). Plant extracts also have great potential, and rutin and mulberry can enhance the thermogenesis of fat by increasing the number of mitochondria (31). Resveratrol increases BAT thermogenesis by increasing SIRT1 (73, 98, 99). These methods are more direct and targeted. BAT transplantation is also a positive approach to anti-obesity (27, 115). It promotes the secretion of certain factors to activate endogenous BAT, and may not only reverse obesity and type 2 diabetes but may also ameliorate PCOS (117). However, clinical applications would require much more research.

## 9 Perspective

Over the last decade, increased BAT activity has garnered great interest, as numerous studies have established an association between BAT activity and metabolic health. Activating BAT or promoting browning can improve glycolipid metabolism and glucose homeostasis (27–29), and transplanting BAT can also improve PCOS (117). Unfortunately, most of the experimental studies are carried out on model animals. Based on the current technology and ethics, it is not realistic to carry out brown fat transplantation surgery on humans. We need to pay more attention to methods that can increase the secretion of brown fat in the body, which seems more practical. BAT activity and body metabolism can be influenced in many ways and through different pathways (**Table 4**). Yet, further study is needed to understand what can effectively, accurately, and safely increase the activity of BAT. The SN, as part of the vegetative nerve, maintains the balance of the body, along with the parasympathetic nerve. When the body is in a state of tension, sympathetic activity plays a major role. The stimulation of sympathetic nerves can cause abdominal visceral cardiovascular contraction as well as heartbeat enhancement and acceleration. Increased sympathetic excitability can cause heartbeat acceleration, and the faster the heartbeat associated with the sympathetic nerve, the higher the cardiovascular mortality (42, 144, 146, 147). Many methods of stimulating the thermogenesis of fat can function through the SNS. In increasing BAT activity, exercise and cold stimulation can significantly activate the SNS (53, 148–150), but much additional research is needed to understand what type of exercise is needed and of what intensity, as well as long it would take to effectively increase BAT activity. Thus, exercise and cold stimulation may not be a good approach for people with cardiovascular disease. Each method can improve BAT activity along a variety of pathways, so it is necessary to evaluate what methods are most suitable for different groups of people. By contrast, it appears to be more versatile and safer to improve BAT activity through the promotion of batokine secretion or through the action of gut microbiota. In addition, studies have shown that these approaches can lead to phenotypes that improve metabolism, anti-obesity, and increased BAT activity, but few studies have demonstrated the causality between these phenotypes, and direct evidence was required to clarify the targets of these methods. Validation with UCP1-KO mice or direct targeting of brown fat would increase the persuasiveness of this perspective. Some views hold that the change of UCP1 mRNA level does not generate heat (151), and it requires activation of β-adrenergic receptors to become active (152). In addition, the feeding environment in animal experiments is basically 22-26°C, which is not a thermoneutrality, which makes the sympathetic nerve of experimental animals may be in a chronic activation state for a long time, thus affecting energy consumption. In view of this, some scholars recently raised mice at 30°C(thermoneutrality) when exploring the impact of time-restricted feeding on energy consumption of mice. This temperature condition is defined as the minimum energy consumption of mice when maintaining body temperature (133). Considering that the amount of active BAT in the human body gradually decreases with age (16), this makes it fundamentally difficult to activate BAT in adults or older people. A large number of studies have shown that the browning of WAT can improve glucose and lipid metabolism and insulin resistance in obese and type 2 diabetes patients (153, 154). The role of browning of WAT is even more important for the prevention and treatment of obesity in human (155). This review highlights the targets of different activation methods and other effects they bring. Regardless, in many methods of activating BAT or promoting browning, we need to choose an efficient, reliable, and safe approach.

## Author contributions

XL provided the idea of this manuscript, ZZ wrote the content, YS was responsible for the collection of materials, HX and MD were responsible for the revision of the article. All authors contributed to the article and approved the submitted version.
